# Food Involvement, Food Choices, and Bioactive Compounds Consumption Correlation during COVID-19 Pandemic: How Food Engagement Influences Consumers’ Food Habits

**DOI:** 10.3390/nu14071490

**Published:** 2022-04-02

**Authors:** Chiara Medoro, Marta Cianciabella, Massimiliano Magli, Giulia Maria Daniele, Nico Lippi, Edoardo Gatti, Roberto Volpe, Vincenzo Longo, Filomena Nazzaro, Silvia Mattoni, Federica Tenaglia, Stefano Predieri

**Affiliations:** 1 Institute for BioEconomy, National Research Council (CNR), Via Piero Gobetti 101, 40129 Bologna, Italy; chiara.medoro@ibe.cnr.it (C.M.); massimiliano.magli@ibe.cnr.it (M.M.); giuliamaria.daniele@ibe.cnr.it (G.M.D.); nico.lippi@ibe.cnr.it (N.L.); edoardo.gatti@ibe.cnr.it (E.G.); stefano.predieri@ibe.cnr.it (S.P.); 2 Health and Safety Unit (SPP), National Research Council (CNR), Piazzale Aldo Moro, 7, 00185 Roma, Italy; roberto.volpe@cnr.it; 3 Institute of Agricultural Biology and Biotechnology (IBBA), National Research Council, 56124 Pisa, Italy; vincenzo.longo@ibba.cnr.it; 4 Institute of Food Sciences, CNR-ISA, Via Roma 64, 83100 Avellino, Italy; filomena.nazzaro@isa.cnr.it; 5 Public Relations Unit, National Research Council (CNR), Piazzale Aldo Moro, 7, 00185 Roma, Italy; silvia.mattoni@cnr.it; 6 Department of Biology, Agriculture and Food Sciences-DiSBA, National Research Council (CNR), Piazzale Aldo Moro, 7, 00185 Roma, Italy; federica.tenaglia@cnr.it

**Keywords:** food involvement scale, food habits, food quality, bioactive compounds, COVID-19, probiotics, efficacy against COVID-19

## Abstract

The containment measures due to the COVID-19 pandemic affected food-related activities, influencing dietary behavior, food habits, and dietary choices. This study aimed to compare the relationship between food involvement and dietary choices before and during the pandemic, investigating the role played by food in dietary habits. Responses given by 2773 Italian consumers to an online survey were studied through the Food Involvement Scale (FIS) and correlated to eating habits. FIS scores were then used to explain the importance given to food in circumstances related to well-being, health, and protection against COVID-19 and used to study the relationship between FIS and bioactive compound knowledge, use, and efficacy against COVID-19. The consumers more involved in food issues recognized the importance of food in circumstances related to well-being, health, and protection against COVID-19 and improved their diet during the pandemic. Moreover, consumers who gave more importance to food also revealed higher attention to the use of healthy substances, such as bioactive compounds, considering them effective against COVID-19. These results showed that food experiencing and involvement could be important elements to promote healthy dietary habits that are essential to maintain physical and mental health during emergency periods such as the COVID-19 pandemic.

## 1. Introduction

Due to the COVID-19 pandemic, many countries around the world decided to limit the virus circulation through containment measures and nationwide lockdown. The restrictions implemented social distancing at different degrees; in Italy, from the beginning of March 2020, the government banned social events and people had to stay at home, and the shops closed except for groceries. Due to the government decree restrictions, there were radical changes in population lifestyles, with a drastic reduction of any form of socialization [1]. In Italy, phases of tougher restrictions interchanged to a progressive reopening of business and social activities following the pandemic curve course from March 2020 through all of 2021 [2]. Social distancing and isolation strongly affected citizens’ everyday behaviors and also affected eating habits and food practices [1,3]. Both the availability of food and the closure of restaurants and catering facilities caused a shift in meal preparation and dietary habits [4]. The increased time spent at home gave a chance to concentrate on meal preparation [3], overcoming barriers that previously caused a decline in home cooking [5] because of lack of time [6]. More time was dedicated to cooking and food skills, as confirmed by web search tools for the word “recipe” [1] indicating an increased involvement in food-related activities. The consumption of homemade dishes has been associated with positive outcomes on health, such as higher consumptions of fruit and vegetables that in turn was related to well-being [3]; on the other hand, stress and depressive symptoms caused by isolation led to a higher consumption rate of “comfort food” (salty snacks and sweets) and the development of dysfunctional eating behaviors [1,4].

People’s interest and care about food vary across socio-demographic features, and it is a subjective characteristic [7,8]. Food engagement can influence dietary behavior and food habits such as food excessive consumption or reduction [9]. Previous research has shown that higher food involvement was related to new food experiences, higher sensitivity [10], higher interest in food sensory properties [7], and most of all, to healthier food choices [11,12]. Several studies measured food involvement so far [13,14], and a general involvement calculation approach was the one offered by the Food Involvement Scale, which takes into account preparation, cooking, and disposal, showing consumers’ sensitivity to food and their experiences, knowledge, and skills [9]. Such a general scale, not related to a specific item or brand, can reveal healthier behaviors but can also highlight negative dietary choices [15].

Studies during the pandemic period showed an increasing interest in a balanced and healthy diet, which could be a preventive action against the clinical outcome of the COVID-19 disease [16], and they focused on health and natural content that were central food choice motives during the COVID-19 pandemic [16].

Thus, the aim of this study was to highlight the relationship between food involvement and dietary choices during the COVID-19 pandemic compared to the pre-COVID period. Furthermore, the research analyzed consumers’ approaches towards functional foods and bioactive compounds, which may play an important role in physical and psychological well-being during the COVID-19 pandemic. Bioactive compounds can be defined as nutrients and non-nutrients present in the food matrix (vegetal and animal sources) that can produce physiological effects beyond their classical nutritional properties [17]. 

During the pandemic period, many consumers looked for additional health protection through the consumption of functional foods and dietary supplements that may have beneficial effects against the COVID-19 disease acting as “immune boosters”. A sales boom in different countries around the world from May 2020 [18] also confirmed the increased interest in bioactive compounds. Bioactive compounds’ efficacy against COVID-19 was highly investigated during the period 2020–2022, as highlighted by the most cited article for each compound: resveratrol [19], lactoferrin [20], quercetin [21], prebiotics [22], probiotics [23], supplements [24], vitamins [25], propolis [26], micronutrients [27], polyphenols [28], vitamin C [29], vitamin D [30], curcumin [31], green tea [32], melatonin [33], vitamin A [25], fibers [28], and fermented food [34].

The present study investigated the role played by food involvement on dietary food habits during the pandemic period in a study population of Italian adults, taking into account the consumer approach towards both food and bioactive compounds.

## 2. Materials and Methods

### 2.1. Study Design and Population

The present study was conducted using an online questionnaire based on the LimeSurvey web app. Data collection was carried out in Italy in April–May 2021 after the first pandemic year. Italian adults aged from 21 to 65 were recruited through social networks, newsletters, and word of mouth. A dedicated webpage, “scienzaatavola.cnr.it”, was created in order to facilitate data collection. Due to social distancing limitations, online tools were chosen, in place of face-to-face interviews, as the easiest and smartest way to recruit respondents during this period. A total of 2773 participants answered the online questionnaire. Characteristics of the population analyzed are listed in Table 1.

All the participants answered the questionnaire voluntarily; they were informed of the main research outcomes and gave consent for their data to be used. Participation in the research was voluntary, and the right to privacy and data protection was respected in accordance with current legislation (GDPR 2016/679).

### 2.2. Survey Measures

The survey was structured in different sections to explore the consumers’ food habits, food involvement, and bioactive compound knowledge and consumption in order to describe the consumers’ habits background after one year of pandemic restrictions.

#### 2.2.1. Socio-Demographic Data and General Habits

This section gathers socio-demographic information such as age (21–40, 41–65), gender, occupation, and Italian region of origin. This section also includes information on habit changes during the COVID-19 pandemic compared to the pre-COVID-19 period. People were asked to quantify their habits changes using a nine-point scale, from “extremely less” (−4), “no changes” (0), to “extremely more” (+4) than the pre-COVID-19 period. Questions on changes were about food practices (food quality, quantity of food eaten at different meals, time spent cooking and eating) and general habits (physical activity, weight gain). Respondents also indicated on a seven-point scale how economic and health prevention factors influenced changes in food habits during the pandemic period from “no changed at all” (1) to “highly changed” (7).

#### 2.2.2. Food Importance

The importance given to food in circumstances related to well-being, health, and protection against COVID-19 was assessed through a seven-point scale from “not important at all” (1) to “very important” (7). Respondents were asked to rate what is food’s importance in maintaining mental skills, physical energy, and serenity and in strengthening the immune system. Moreover, the importance given to food was also assessed in restraining and controlling COVID-19 infection and in recovering from COVID-19 disease and its vaccine side effects.

#### 2.2.3. Dietary Habits before and during the COVID-19 Pandemic

In order to assess respondents’ dietary habits before and during the pandemic period, people were asked to indicate the consumption frequency of different food types before the COVID-19 pandemic using a six-point scale from “never” (1) to “several ratios per day” (6). 

Food consumption variation during the pandemic was rated using a nine-point scale from “extremely less” (−4), “no changes” (0), to “extremely more” (+4) than the pre-COVID period for each food type.

#### 2.2.4. Food Involvement Scale

Food involvement, defined as the importance level given to food, was measured through the Food Involvement Scale. The FIS consisted of two subscales: set and disposal (SD) and preparation and eating (PE) [35]. FIS took into account five different stages of food involvement: acquiring, preparing, cooking, eating, and disposal. Factor analysis indicated individual scale items loads onto SD and PE [36]. Participants were asked to rate the extent to which they agreed to 12 items on a seven-point scale from “strongly disagree” (1) to “strongly agree” (7). The questionnaire items measured the characteristics of food involvement based on activities related to food acquisition, preparation, cooking, eating, and disposal [7]. The individual score for each subscale was computed as the sum of ratings given to the statements, with higher scores reflecting higher involvement levels [35]. The scale could range from a low score of 12 to a high score of 84 [7]. The FIS score was divided into three groups (low, medium, and high FIS) according to the tertile distribution, including 33% of each sample. The lowest tertile represented the “low” involvement group, while the highest tertile was the “high” involvement one [7].

#### 2.2.5. Bioactive Compounds and Their Role in the COVID-19 Pandemic

This section was about the bioactive compound knowledge level and their importance against COVID-19.

Respondents were presented with two items for each substance type. 

First, they were asked to indicate if they know and use the bioactive compound through a four-point scale, and the available answers were: “I have never heard about it”, “I know it but I do not care about it”, “I know it and I would like to use it”, and “I know it and I am using it”. In each answer, three binary factors (0, 1) related to “use”, “knowledge”, and “interest” were considered. Thus, the resulting score was computed as the sum of ratings given to the three factors: “I have never heard about it”(0, 0, 0); “ I know it but I do not care about it”(0, 1, 0); “I know it and I would like to use it”(0, 1, 1); “I know it and I am using it”. (1, 1, 1).

Finally, for each bioactive compound, it was also asked if it was considered effective against COVID-19 infection.

### 2.3. Statistical Analysis

Data were analyzed using IBM SPSS V. 26. Descriptive statistics were used for socio-demographic data. 

A two-way analysis of variance was used to determine the main effects of gender (male, female) and age (21–40; 41–65), and their interaction, on FIS score and its subscales (SD and PE).

F-value was used to determine whether the between-group variability of means was larger than the within-group variability of the individual values.

Pearson’s correlation coefficients were computed for an overall view of the relationships between general habits, factors influencing changes in food habits, and FIS score.

Three-way analysis of variance was used to determine the main effect of FIS groups (low, medium, and high), gender (M, F,), and age (21–40; 41–65) with interactions (FIS*Gender and FIS*age). Tuckey’s post hoc test was also used to test the differences between the FIS groups. Differences were considered significant when *p* < 0.05.

Bioactive compound efficacy was analyzed through a contingency table analysis; a chi-square test was performed to measure associations between FIS groups and efficacy for each bioactive compound.

## 3. Results

### 3.1. Socio-Demographic Data

There were 2773 study participants, with a prevalence of women (64.6%) compared to men (35.4%), equally distributed in two age classes (21–40, 41–65). The region of origin most represented was Northern Italy, but respondents came from all Italian regions. Most of the respondents were employed (62.3%), and some of them were students (25.9%). The sample characteristics are summarized in Table 1.

### 3.2. FIS Data Analysis

Mean values for the FIS score and its subscales, set and disposal (SD) and preparation eating (PE), were computed for the whole population considered. The results are listed in Table 2.

FIS score was mainly influenced by gender; higher ratings were registered for females than males for both FIS and its subscales (SD and PE). The age influenced only the SD scale, with the older group (41–65) having a higher SD rate than the younger ones.

### 3.3. General Habits

General habit changes such as food quality, quantity of food eaten at different meals, time spent cooking and eating, physical activity, and weight gain were summarized (data not shown) and correlated. FIS score was also included in the correlation analysis to complete the general population background. The results are listed in Table 3.

The table also includes habit changes due to health prevention and economic factors during the COVID-19 pandemic.

FIS score was positively correlated with the increase of food quality and time dedicated to cooking, and eating, moreover, was also slightly correlated to the amount of food eaten at lunch and voluntary changes in food habits for health prevention. No correlations were found between FIS and changes in physical activity and weight. Physical activity was negatively correlated with weight gain and food amount eaten for lunch, dinner, and snacks, while positive correlations were found with food amount eaten for breakfast, time dedicated to eating, and voluntary changes in food habits to support health. Weight gain, besides the negative correlation with physical activity, was correlated with food consumption increase for all the meals (breakfast, lunch, dinner, and snacks), time dedicated to cooking and eating, and to changes in economics. Besides affecting weight gain, economic changes were correlated to reduced food quality and amount of food. Voluntary changes in food habits for health prevention purposes were positively correlated to improved food quality, increased time dedicated to cooking, and physical activity, while the correlation with food quantity eaten at lunch, dinner, and snacks decreased. 

### 3.4. Food Importance

Differences in the importance given to food in situations related to well-being and protection against COVID-19 were analyzed by age, gender, and FIS group and are summarized in Table 4.

The high FIS group gave the highest importance to food compared to the medium and low groups that gave, respectively, medium and low importance. High FIS respondents considered food helpful to strengthen the immune system and maintain physical energy, mental skills, and serenity, while recovering from illnesses; moreover, they also considered food effective in controlling COVID-19 infection, restraining COVID-19 infection effects, recovering from COVID-19, and mitigating vaccine side effects. Interactions with gender and age can be considered of no relevance since they showed low F values compared to the one registered for the FIS score.

### 3.5. Food Importance

Differences in food consumption in the pre-COVID period were analyzed by FIS score groups, age, and gender since there was a higher number of women. The results are shown in Table 5.

Respondents with high FIS showed a higher consumption frequency of fish, vegetables, fruit, and eggs, while low FIS consumers had lower consumption of those foods; the medium FIS group had intermediate frequency consumptions. Gender discrepancy, as well as age, did not affect the consumption of those foods. Differences in meat consumption were mainly related to gender, with males having higher meat consumption than women. A tendency to be significant (*p* < 0.10) was also observed for bread and pasta, consumed more by the high FIS group than the low FIS group. Younger people (21–40) seemed to prefer bread and pasta more than people of medium age (41–65), and this variation did not influence differences in FIS group consumption frequency. Cheese consumption differences were mainly related to gender, with males consuming more cheese than women did.

The food consumption variation during the pandemic period is presented in Table 6. The high FIS group increased the consumption of fish, legumes, eggs, fruit, citruses, vegetables, rice, and organic food, while it decreased the use of sparkling drinks to a higher extent compared to the medium and low groups. A slight consumption increase for those foods was also registered for medium and low FIS, except for eggs and organic food, for which the consumption seemed to decrease only for low FIS. Gender and age influences can be considered of no relevance as confirmed by F values.

Interestingly, a pasta consumption increase was only related to men, as observed for dairy products. Women decreased more beer and wine consumption as well as sauces.

Differences related only to age were registered for spirits and food for intolerances, for which the consumption decrease was higher in the younger group. Younger people also declared to increase light food use compared to people in the 41–65 age class, who decreased their use. 

### 3.6. Bioactive Compounds and Their Role in the COVID-19 Pandemic

Table 7 summarizes the differences in nutraceutical awareness by age, gender, and FIS score. Ratings closer to 0 mean poor knowledge of the substance, and values closer to 3 mean awareness and use of the substance.

High FIS respondents were more aware and declared to use bioactive compounds such as lactoferrin, prebiotics, probiotics, supplements, vitamins (particularly vitamins A, C, and D), propolis, micronutrients, polyphenols, curcumin, melatonin, and fibers. Among those compounds, lactoferrin was the least known substance, with ratings close to 0 for all the FIS groups, while melatonin was the most used by the high FIS group, and polyphenols were the ones showing a clear discrepancy between the high FIS and low FIS groups, which showed a value close to 0, indicating poor knowledge of the substance.

Medium FIS respondents showed intermediate knowledge levels, while the low FIS were the least aware group. Gender and age discrepancies did not affect FIS group differences, as confirmed by *p* and F values.

Among all of the bioactive compounds considered, resveratrol, quercetin, and fermented cereals showed ratings around 0, and the differences only related to age; respondents aged 41–65 were more aware of those substances. According to age differences, probiotics were the most known and used by the 41–65 age class.

Differences related to gender showed that women consumed more green tea than men did.

Bioactive compounds’ efficacy against COVID-19 was also asked, and the results analyzed by FIS score are listed in Table 8.

Most of the respondents seemed not to know the topic. According to the FIS score, the high FIS group considered nutraceuticals more effective against COVID-19 than low FIS respondents did. The medium FIS respondents showed intermediate trends.

Among the bioactive compounds considered, melatonin showed an opposite trend, with the low FIS considering it more effective than the high FIS did, while there were no differences between high and low FIS about the fermented cereals.

Differences between FIS groups were statistically significant only for the following substances: lactoferrin, prebiotics, probiotics, propolis, vitamins C and D and other vitamins.

Results on the efficacy against COVID-19, according to consumers’ consensus, were used to compare with the number of publications on the bioactive compounds’ applications against COVID-19, thus giving an insight into the public’s understanding of scientific information. As shown in Table 9, there was a correspondence between the consumers’ opinion and the interest of the scientific community, represented by the number of publications. Vitamins were the most studied against COVID-19 and the most effective, according to consumers. Exceptions were represented by propolis and prebiotics, which are considered effective by a considerable number of consumers but not by scientific research, which dedicated few articles to them.

## 4. Discussion

This study provided a description of the eating habits and lifestyle changes after one year of the COVID-19 pandemic in Italy, focusing on the importance given to food in the dietary choices and in nutraceuticals knowledge and intake. FIS ratings, analyzed in the whole population during the COVID-19 pandemic, showed women’s higher food involvement compared to men, confirming what was previously stated by Bell and Marshall [7]. Social, psychological, and cultural reasons accounted for the differences between genders. Women traditionally took care of the meal preparation, resulting in higher involvement in healthy dietary choices for themselves and their family members [37]. Moreover, women’s attention to healthy food [38,39] explained higher FIS ratings; as shown in previous studies [7,8,9,10,11,12,13,14,15,16,17,18,19,20,21,22,23,24,25,26,27,28,29,30,31,32,33,34,35,36,37,38,39,40], people highly involved with food also choose healthy diets. Age also played a role in the food involvement to some extent, particularly in the meal set and disposal; higher SD values were registered for the older age group (41–65). Indeed, in older age, a greater value was recognized for food preparation since it was related to positive social relationships and a supportive social network [41]. 

In this study, FIS score correlations highlighted the influence of food importance on general habits during the pandemic. Results that were obtained showed that consumers who were more involved with food paid more attention to food quality, the quantity of food eaten for lunch, time spent for cooking and eating, and changes in their food habits for health prevention purposes rather than economic reasons. These findings are in line with what was observed in other studies [42]. During the pandemic in Italy, consumers having a healthy diet paid more attention to food quality and were the keenest to follow dietary recommendations for health purposes. On the other hand, consumers who followed an unhealthy diet paid more attention to prices and showed economic problems. Results related to physical activity showed that it was associated with a healthier lifestyle, a reduction of food eaten at lunch, dinner, and snacks, an increase of food eaten for breakfast, and more attention paid to their diet in order to support their health. On the other hand, weight gain, negatively correlated to physical activity, was associated with worse habits, food consumption increase at all meals, and more time spent cooking and eating. The correlation with dietary changes due to economic reasons confirmed that economic difficulties were often coupled with diet deteriorations [42]. These results confirmed the observations by Bennet et al. [43], that COVID-19 had both positive impacts on dietary habits, such as the consumption of fresh products, and negative effects, such as poor lifestyle habits (weight gain, limited physical activity, and mental health issues). 

The importance given to food in situations related to well-being and protection against COVID-19 was higher in high FIS respondents than in medium and low FIS groups. The high FIS group considered food helpful to strengthen the immune system and to maintain physical energy, mental skills, and serenity while recovering from illnesses, especially from COVID-19 and its vaccine side effects; moreover, they also considered food effective in controlling COVID-19 infection. These results are in accordance with food choice motive changes highlighted during the COVID-19 pandemic in Europe [44]; health and mood became the main food choice motives during the lockdown, and people more motivated by health also adopted healthier diets [45].

Differences in food consumption in the pre-COVID period showed that the high FIS group consumed more fish, vegetables, fruits, and eggs, while the low FIS consumers had lower consumptions of these foods; the medium FIS group had intermediate frequency consumptions. Indeed, consumers with high food involvement were reported to follow healthy dietary practices, using more vegetables and being more engaged in meal preparation from scratch [45], which could explain the high consumption of eggs, which are used for many meal preparations and for, often homemade, bread and pasta. Gender discrepancy, as well as age, did not affect the consumption of these foods.

Differences related to gender confirmed that men were more likely to eat high-fat food [15], showing a higher consumption of cheese and meat. Younger people (21–40) seemed to prefer bread and pasta more than people of medium age (41–65), as was already observed in Northern Europe by Bamia et al. [46].

During the pandemic period, food consumption variation confirmed trends observed in the pre-COVID time. The high FIS increased, to a higher extent, the use of fish, legumes, eggs, fruits, vegetables, rice, and organic food, while decreasing the use of sparkling drinks. A slight consumption increase for those foods was also registered for medium and low FIS, except for eggs and organic food, for which the consumption seemed to decrease only for low FIS. These results are in accordance with other studies conducted in Italy during the pandemic that highlighted a consumption increase of vegetables, legumes, fruits, and fish and a consumption decrease of snacks and carbonated drinks in people with a high adherence to the Mediterranean diet [4,42]. The same quality increase was also observed in other countries following the Mediterranean diet [47]. Indeed, the main novelty of the present research was to highlight the correlation between food involvement and diet quality during the COVID-19 pandemic.

Interestingly, a pasta consumption increase was only related to men as for dairy products, as previously confirmed by Wirfält et al. [48]. Women decreased more beer and wine consumption as well as sauces, confirming women’s strong belief in healthy eating [38,39].

Differences related only to age were registered for spirits and food for intolerances, for which the consumption decrease was higher in the younger group. Indeed, alcohol consumption is mainly related to young people’s nightlife and social occasions [49], which were limited during the pandemic period, while being at home may have changed the use of food for intolerance since people were able to choose and cook tailored raw food. Younger people also declared to increase light food use compared to people in the 41–65 age class, which could be related to increased attention towards weight gain determined by the confinement.

As observed with healthy food, the association between food involvement and bioactive compound consumption and awareness during the pandemic period revealed that high FIS respondents were more aware and declared to use bioactive compounds such as lactoferrin, prebiotics, probiotics, supplements, vitamins (particularly vitamins A, C, and D), propolis, micronutrients, polyphenols, curcumin, melatonin, and fibers. Micronutrients (zinc, iron, magnesium, selenium, and copper), known for having an effective role in protection against viral infections [27], were also well known by the respondents. Lactoferrin was the least known substance among the three FIS groups, while melatonin was the most used by high FIS, and polyphenols were the ones showing a clear discrepancy between high FIS and low FIS, who showed poor knowledge of the substance. Again, people who gave more importance to food revealed a higher attention to a healthy diet and were more inclined towards new food experiences [10], such as new substances useful for health, as bioactive compounds are for most of the consumers [50]. Moreover, the high FIS, more involved in cooking, were aware of the food properties of common foods such as oil, which is rich in polyphenols. The low FIS group, which showed less interest and knowledge of bioactive compounds, were the ones following an unhealthy diet during the pandemic, which was associated with socio-economic problems [51,52]; indeed, the high cost was reported to be one of the barriers of using nutraceuticals [50].

Among all of the bioactive compounds considered, resveratrol, quercetin, and fermented cereals were the least known, but respondents aged 41–65 were more aware of those substances. Resveratrol’s health properties were identified and described for more than two decades [53]; the association of this substance with a key Italian drink, which contained it, such as wine [54], can explain its familiarity among adults. The same can be supposed for quercetin [55]. The awareness of fermented cereals among adults can be related to their role in decreasing the risk of age-related diseases, such as cardiovascular diseases [56]. According to age differences, probiotics were the most known and used by the 41–65 age class, as they are commonly used to slow age-related changes [57,58].

Differences related to gender showed that women consumed more green tea than men did, despite what was observed by Shimbo et al. [59].

Results on bioactive compounds’ efficacy against COVID-19 showed that most of the respondents did not know the topic. Indeed, among consumers, there was a great lack of knowledge about nutraceuticals and their benefits [50]. The high FIS group considered bioactive compounds more effective against COVID-19 than low FIS respondents did. The medium FIS respondents showed intermediate trends. This was not unexpected, since perceived health benefits was one of the drivers for nutraceuticals intake [60,61], and consumers more aware of health benefits were motivated to take nutraceuticals [62,63], as the high FIS group showed, while those who doubted the health benefits avoided nutraceuticals intake [64,65], such as for medium and low FIS respondents.

The opposite trend showed by melatonin, with the low FIS considering it more effective than the high FIS did, could be related to its chronobiotic function, regulating sleep, energy metabolism, and anti-inflammatory effects [66]. Bad dietary habits, as the low FIS group showed, were associated with sleep disorders [67]; thus, low FIS could be more interested in melatonin and trust its beneficial effects.

The comparison between the efficacy against COVID-19, according to the consumers’ consensus and the number of publications on the bioactive compounds’ applications against COVID-19, showed that there was a general correspondence between the public’s understanding and the scientific information, with some exceptions. Vitamins were the most studied against COVID-19 and the most effective, according to consumers. While the poor interest of the scientific community on propolis and prebiotics was not in line with the consumers’ expectations, indicating scientific misinformation and miscommunication and confirming what was already observed by Teoh et al., there is a lack of knowledge and clinical evidence on nutraceuticals, which also affects consumption [50].

The main novelty of this research was dealing with the food involvement influence on dietary habits and bioactive compounds intake during the COVID-19 pandemic. So far, this was the first study analyzing such a correlation during the pandemic by using the FIS scale.

An additional advantage of this study was the timing of the survey that was launched after one year of the COVID-19 pandemic, so that the data collected gave an overview of how consumers adapted to the emergency.

## 5. Conclusions

This study provided a description of the eating habits and lifestyle changes after one year of the COVID-19 pandemic in Italy, focusing on the importance given to food in the dietary choices and in the bioactive compound knowledge and intake.

The results showed that food engagement had an impact on dietary habits during the COVID-19 pandemic. Consumers more involved in food paid more attention to food quality, the quantity of food eaten for lunch, time spent cooking and eating, and changes in their food habits for health prevention purposes. Moreover, they recognized the importance of food in circumstances related to well-being, health, and protection against COVID-19.

In the present research, trends observed during the COVID-19 pandemic confirmed what was already described in the pre-COVID period; consumers more involved in food improved their diet quality, increasing the consumption of healthy food such as fish, legumes, eggs, fruits, vegetables, rice, and organic food, while they reduced carbonated drinks.

People who gave more importance to food paid higher attention not only to a healthy diet but also to the use of healthy substances such as bioactive compounds. Consumers highly involved in food showed to know better about and use more bioactive compounds, which were also considered effective against COVID-19 infection. The consumers’ consensus on the efficacy was in line with scientific research interests, with some exceptions that showed a need for improving the communication of the scientific research outcomes.

Since respondents were asked to give information retroactively, a possible limitation could be a recall bias due to the chance of providing inaccurate estimates. Such a limitation was unavoidable since the pandemic duration was unpredictable, and it was impossible to organize a prospective data collection. Another limitation is related to the population composition being mainly composed of adults and women, thus making it difficult to use this data for research studies on other age categories. As this study was conducted through an online-based survey with anonymous participation, it was impossible to exclude such information reliability bias a priori. Therefore, the statistical data verification process was performed to avoid this bias as much as possible. 

This research gives an overview of the dietary habits during the COVID-19 pandemic and can be a relevant element for future public health policy actions; food experiencing and involvement could induce more effective healthy dietary changes, essential to maintain physical and mental health during emergency periods such as the COVID-19 pandemic. Moreover, this study showed that scientific information could collide with the public’s understanding. The scientific community should make an effort to increase the knowledge about bioactive compound efficacy, improving communication on scientific and clinical evidence that could promote bioactive compound intake, exploiting the sensitivity associated with food involvement.

## Figures and Tables

**Table 1:** General characteristics of the study participants (*n* = 2773).

	*n*	%
Gender		
Female	1790	64.6
Male	983	35.4
Age (years)		
	21–40	48
	41–65	52
Geographic area		
North west	858	30.9
North east	879	31.7
Center	498	18.0
South and islands	538	19.4
Occupation		
Unemployed	232	8.4
Employed	1727	62.3
Retired	61	2.2
Student	718	25.9
Missing	35	1.3

**Table 2:** Two-way ANOVA model on FIS score, SD score, and PE score with an interaction (fixed factors: gender and age) for the whole population considered. The FIS scale ranged from 12 to 84.

	Gender				Age				Gender × Age	
	Female	Male	*p*	F	21–40	41–65	*p*	F	*p*	F
FIS score	63.3	59.7	<0.001	87.5	61.4	61.7	0.423	0.7	0.122	2.4
SD	16.7	15.4	<0.001	103.3	15.7	16.4	<0.001	31.4	0.971	0.00
PE	46.6	44.3	<0.001	48.3	45.7	45.3	0.209	1.6	0.073	3.2

**Table 3:** Pearson’s correlation coefficients on general habit changes during the COVID-19 pandemic and FIS score. * *p* < 0.05 level; ** *p* < 0.01.

	FIS Score	Physical Activity	Weight Gain	Food Quality	Food Amount (Breakfast)	Food Amount (Lunch)	Food Amount (Dinner)	Food Amount–(between Meals)	Cooking Time	Eating Time	Food Changes for Prevention	Changes Due to Economics
FIS score	1	0.03	0.02	0.16 **	0.03	0.06 **	0.02	−0.01	0.23 **	0.13 **	−0.02	0.05 *
*p*		0.112	0.269	<0.001	0.113	0.004	0.351	0.701	<0.001	<0.001	0.267	0.016
Physical activity	0.03	1	−0.28	0.13 **	0.05 **	−0.08	−0.09	−0.08	0.03	0.07 **	−0.02	0.09 **
*p*	0.112		<0.001	<0.001	0.006	<0.001	<0.001	<0.001	0.180	0.001	0.308	<0.001
Weight gain	0.021	−0.28	1	−0.02	0.06 **	0.24 **	0.26 **	0.18 **	0.08 **	0.04 *	0.07 **	0.01
*p*	0.269	<0.001		0.222	0.003	<0.001	<0.001	<0.001	<0.001	0.023	<0.001	0.527
Food quality	0.155 **	0.13 **	−0.02	1	0.12 **	0.12 **	0.08 **	0.02	0.36 **	0.29 **	−0.12	0.15 **
*p*	<0.001	<0.001	0.222		<0.001	<0.001	<0.001	0.309	<0.001	<0.001	<0.001	<0.001
Food amount (breakfast)	0.03	0.05 **	0.06 **	0.12 **	1	0.34 **	0.27 **	0.20 **	0.12 **	0.07 **	−0.02	0.03
*p*	0.113	0.006	0.003	<0.001		<0.001	<0.001	<0.001	<0.001	<0.001	0.214	0.094
Food amount (lunch)	0.06 **	−0.08	0.24 **	0.12 **	0.34 **	1	0.52 **	0.36 **	0.14 **	0.15 **	−0.06	−0.09
*P*	0.004	<0.001	<0.001	<0.001	<0.001		<0.001	<0.001	<0.001	<0.001	0.003	<0.001
Food amount (dinner)	0.02	−0.09	0.26 **	0.08 **	0.27 **	0.52 **	1	0.39 **	0.10 **	0.11 **	−0.02	−0.07
*p*	0.351	<0.001	<0.001	<0.001	<0.001	<0.001		<0.001	<0.001	<0.001	0.396	<0.001
Food amount–(between meals)	−0.01	−0.08	0.1813 **	0.02	0.20 **	0.36 **	0.39 **	1	0.06 **	0.08 **	−0.08	−0.09
*p*	0.701	<0.001	<0.001	0.309	<0.001	<0.001	<0.001		0.001	<0.001	<0.001	<0.001
Cooking time	0.225 **	0.03	0.08 **	0.360 **	0.12 **	0.14 **	0.10 **	0.06 **	1	0.30 **	0.02	0.13 **
*p*	<0.001	0.180	<0.001	<0.001	<0.001	<0.001	<0.001	0.001		<0.001	0.384	<0.001
Eating time	0.13 **	0.06 **	0.04 *	0.29 **	0.07 **	0.15 **	0.11 **	0.08 **	0.30 **	1	−0.08	0.01
*p*	<0.001	0.001	0.023	<0.001	<0.001	<0.001	<0.001	<0.001	<0.001		<0.001	0.450
Food changes for prevention	−0.02	−0.02	0.07 **	−0.12	−0.02	−0.06	−0.02	−0.08	0.02	−0.08	1	0.35 **
*p*	0.267	0.308	<0.001	<0.001	0.214	0.003	0.396	<0.001	0.384	<0.001		<0.001
Changes due to economics	0.05 *	0.09 **	0.01	0.15 **	0.03	−0.09	−0.07	−0.09	0.13 **	0.01	0.35 **	1
*p*	0.016	<0.001	0.527	<0.001	0.094	<0.001	<0.001	<0.001	<0.001	0.450	<0.001	

**Table 4:** Three-way ANOVA model on the importance given to food to help recover in some circumstances. FIS group, gender, and age were used as fixed factors with the interactions FIS group × Age and FIS group × Gender. Tuckey’s post hoc test was also used to test the differences between the FIS groups.

Food	FIS Group					Gender				Age				FIS Group × Gender		FIS Group × Age	
	Low	Medium	High	*p*	F	Female	Male	*p*	F	21–40	41–65	*p*	F	*p*	F	*p*	F
Meat	2.6	2.6	2.6	0.764	0.3	2.5	2.7	<0.001	19.2	2.7	2.5	<0.001	20.59	0.378	1.0	0.743	0.3
Fish	1.9 b	2.0 a	2.1 a	<0.001	16.1	2.0	2.8	0.006	7.6	2.0	2.0	0.157	2.00	0.225	1.5	0.517	0.7
Vegetables	3.5 c	3.8 b	4.0 a	<0.001	56.8	4.0	3.6	<0.001	106.5	3.8	3.8	0.897	0.02	0.442	0.8	0.026	3.6
Fruit	3.4 c	3.6 b	3.8 a	<0.001	19.0	3.6	3.6	0.137	2.2	3.5	3.7	<0.001	35.67	0.627	0.5	0.366	1.0
Bread and pasta	3.6	3.60	3.7	0.078	2.6	3.6	3.6	0.244	1.4	3.7	3.5	<0.001	35.58	0.639	0.5	0.132	2.0
Cheese	2.8	2.8	2.8	0.576	0.6	2.8	2. 9	0.001	11.6	2.9	2.8	0.003	8.85	1.030	0.4	0.897	0.1

a, b, c: Means with different letters correspond to statistical differences (*p* < 0.05).

**Table 5:** Three-way ANOVA model on food consumption frequency in the pre-COVID period. FIS group, gender, and age were used as fixed factors with the interactions FIS group × Age and FIS group × Gender. Tuckey’s post hoc test was also used to test the differences between the FIS groups.

Topic	FIS Group					Gender				Age				FIS Group × Gender		FIS Group × Age	
	Low	Medium	High	*p*	F	Female	Male	*p*	F	21–40	41–65	*p*	F	*p*	F	*p*	F
Health improving	5.7 c	6.2 b	6.5 a	<0.001	111.0	6.3	6.0	<0.001	45.4	1	6	0.776	0.1	0.017	4.1	0.505	0.7
Strengthen immune system	5.5 c	5.9 b	6.3 a	<0.001	90.1	6.1	5.7	<0.001	58.7	1	6	0.420	0.7	0.009	4.8	0.304	1191
Illness recovering	5.5 c	5.9 b	6.2 a	<0.001	70.9	6.0	5.7	<0.001	43.2	1.15	6	0.001	12.0	0.047	3.1	0.316	1.2
Maintain physical energy	5.7 c	6.1 b	6.4 a	<0.001	92.7	6.3	5.9	<0.001	63.5	0.30	6	0.030	4.7	0.018	4.0	0.743	0.3
Maintain mental skills	5.5 c	5.9 b	6.3 a	<0.001	121.2	6.1	5.7	<0.001	67.1	1	6	0.103	2.7	<0.001	8.9	0.341	1.1
Maintain serenity	5.5 c	6.0 b	6.4 a	<0.001	147.4	6.1	5.8	<0.001	39.3	2	6	0.671	0.2	0.077	2.6	0.197	1.6
COVID-19 infection control	2.9 b	3.0 b	3.2 a	0.006	5.1	3.1	3.1	0.178	1.8	0.33	3	0.003	8.6	0.534	0.6	0.722	0.3
Restrain COVID-19 infection effects	4.2 b	4.0 b	4.6 a	<0.001	23.7	4.3	4.2	0.049	3.9	4	4	0.011	6.5	0.213	1.6	0.025	3.7
Recover from COVID-19	4.8 c	5.2 b	5.3 a	<0.001	25.1	5.2	5.0	<0.001	12.8	5	5	533	<0.001	0.261	1.3	0.510	0.7
Mitigate COVID-19 vaccine side effects	3.2 b	3.3 b	3.5 a	0.002	6.0	3.3	3.4	0.537	0.4	3	3	3.49	0.002	0.687	0.4	0.548	0.6

a, b, c: Means with different letters correspond to statistical differences (*p* < 0.05).

**Table 6:** Three-way ANOVA model on food variation consumption in the COVID-19 period. FIS group, gender, and age were used as fixed factors with the interactions FIS group × Age and FIS group × Gender. Tuckey’s post hoc test was also used to test the differences between the FIS groups.

Food	FIS Group					Gender				Age				FIS Group × Gender		FIS Group × Age	
	Low	Medium	High	*p*	F	Female	Male	*p*	F	20–40	41–65	*p*	F	*p*	F	*p*	F
Pasta	0.05	0.05	0.00	0.576	0.6	−0.01	0.08	0.048	3.9	0.01	0.07	0.157	2.0	0.976	0.0	0.418	0.9
Bread	0.02	−0.03	0.01	0.608	0.5	−0.02	−0.00	0.842	0.0	0.00	0.01	0.952	0.0	0.577	0.6	0.524	0.6
Meat	−0.13	−0.19	−0.19	0.517	0.7	−0.21	−0.13	0.096	2.8	−0.16	−0.17	0.763	0.1	0.070	2.7	0.757	0.3
Fish	0.04	0.17	0.19	0.030	3.5	0.13	0.14	0.920	0	0.12	0.15	0.615	0.3	2.32	0.1	0.712	0.3
Legume	0.15 c	0.31 b	0.44 a	<0.001	15.0	0.32	0.28	0.441	0.6	0.33	0.26	0.105	2.6	0.553	0.6	0.182	1.7
Dairy products	−0.00	−0.01	−0.02	0.941	0.1	−0.06	0.04	0.015	1.0	−0.04	0.01	0.212	1.6	0.845	0.2	0.211	1.6
Eggs	−0.02 b	0.07 b	0.14 a	0.04	5.7	0.06	0.06	0.999	0.0	0.08	0.06	0.679	0.2	0.490	0.6	0.677	0.4
Fruit	0.35 b	0.44 b	0.61 a	<0.001	12.2	0.45	0.48	0.45	0.5	0.47	0.47	0.982	0	0.008	4.8	0.528	0.6
Citruses	0.12 b	0.26 a	0.34 a	<0.001	9.1	0.22	0.26	0.42	0.7	0.16	0.31	<0.001	12.2	0.031	3.5	0.264	1.3
Vegetables	0.43 c	0.64 b	0.82 a	<0.001	25.5	0.68	0.58	0.023	5.2	0.65	0.61	0.428	0.6	0.137	2.0	0.267	1.3
Beer and wine	−0.40	−0.23	−0.028	0.078	2.6	−0.40	−0.20	0.002	9.2	−0.36	−0.25	0.116	2.5	0.030	1.0	0.96	0.0
Spirits	−0.98	−0.89	−1.06	0.181	1.7	−1.00	−0.95	0.414	0.7	−1.08	−0.88	0.006	7.5	0.815	0.2	0.846	0.2
Coffee	0.12	0.13	0.12	0.989	0.0	0.14	0.11	0.976	0.0	0.12	0.12	0.995	0	0.580	0.5	0.762	0.3
Rice	0.06 b	0.13 b	0.25 a	<0.001	8.0	0.11	0.18	0.077	3.1	0.16	0.13	0.512	0.4	0.437	0.8	0.307	1.2
Sauces	−0.26	−0.26	−0.30	0.752	0.3	−0.36	−0.18	<0.001	15.1	−0.23	−0.30	0.193	1.7	0.843	0.2	0.075	2.6
Organic food	−0.11 b	0.11 b	0.28 a	<0.001	24.2	0.15	0.03	0.014	6.0	0.19	0.01	<0.001	15.4	0.065	2.7	0.963	0.0
Light food	−0.14	−0.07	−0.03	0.143	1.9	−0.01	−0.15	0.003	9.0	0.08	−0.23	<0.001	43.8	0.504	0.7	0.304	1.2
Food for intolerances	−0.26	−0.24	−0.25	0.938	0.1	−0.22	−0.28	0.245	1.4	−0.15	−0.33	<0.001	13.5	0.654	0.4	0.877	0.1
Sparkling drinks	−0.61 b	−0.68 a	0.87 a	0.002	6.0	−0.72	−0.72	0.954	0	−0.72	−0.72	0.998	0	0.536	0.6	0.107	2.2

a, b, c: Means with different letters correspond to statistical differences (*p* < 0.05).

**Table 7:** Three-way ANOVA model on the knowledge level of different bioactive compounds during the COVID-19 period. FIS group, gender, and age were used as fixed factors with the interactions FIS group × Age and FIS group × Gender. Tuckey’s post hoc test was also used to test the differences between the FIS groups.

Substance	FIS Group					Gender				Age				FIS Group × Age		FIS Group × Gender	
	Low	Medium	High	*p*	F	Female	Male	*p*	F	21–40	41–65	*p*	F	*p*	F	*p*	F
Fibers	2.19 b	2.40 a	2.46 a	<0.001	21.10	2.4	2.31	0.013	6.16	2.34	2.36	0.585	2.34	0.106	2.25	0.879	0.13
Vitamins	2.20 b	2.30 ab	2.37 a	0.001	7.30	2.3	2.3	0.039	4.27	2.31	2.27	0.313	1.02	0.0149	1.90	0.082	2.51
Mineral salts	1.99 b	2.13 a	2.22 a	<0.001	11.20	2.11	2.12	0.982	0	2.11	2.12	0.812	2.11	0.506	0.68	0.35	1.05
Supplements	1.90 b	1.98 a	2.08 a	0.001	6.84	2.06	1.91	<0.001	13.00	1.97	2	0.415	1.97	0.788	0.24	0.696	0.36
Probiotics	1.51 c	1.68 b	1.86 a	<0.001	21.50	1.79	1.58	<0.001	20.7	1.59	1.78	<0.001	1.59	0.009	4.68	0.847	0.17
Fermented cereals	1.25 b	1.41 a	1.49 a	<0.001	10.70	1.39	1.38	0.738	0.11	1.42	1.34	0.062	1.42	0.218	1.52	0.112	2.19
Prebiotics	1.20 c	1.31 b	1.51 a	<0.001	16.30	1.41	1.28	0.005	8.05	1.24	1.45	<0.001	1.24	0.109	2.22	0.92	0.08
Polyphenols	0.96 c	1.11 b	1.28 a	<0.001	18.10	1.07	1.17	0.025	5.01	0.97	1.26	<0.001	0.97	0.082	2.50	0.724	0.32
Vitamin C	2.31 b	2.46 a	2.45 a	0.001	7.36	2.41	2.41	0.916	0.01	2.37	2.44	0.065	2.37	0.658	0.42	0.668	0.40
Vitamin D	2.14 b	2.25 a	2.28 a	0.003	5.84	2.34	2.11	<0.001	42.10	2.18	2.27	0.013	2.18	0.937	0.07	0.508	0.68
Vitamin A	1.76 b	1.88 b	1.93 a	0.001	7.06	1.82	1.89	0.07	3.29	1.86	1.86	0.979	1.86	0.194	1.64	0.714	0.34
Green tea	1.94 b	2.14 a	2.23 a	<0.001	18.50	2.14	2.06	0.062	3.50	2.12	2.08	0.364	2.12	0.775	0.26	0.612	0.49
Propolis	1.61 c	1.71 b	1.85 a	<0.001	11.02	1.82	1.63	<0.001	21.50	1.66	1.79	0.004	1.66	0.789	0.24	0.182	1.70
Melatonin	1.52 b	1.57 ab	1.59 a	0.216	1.53	1.65	1.47	<0.001	21.10	1.57	1.55	0.546	1.57	0.996	0	0.906	0.10
Curcumin	1.26 b	1.33 a	1.56 a	<0.001	15.5	1.42	1.35	0.12	2.42	1.25	1.52	<0.001	1.25	0.259	1.35	0.37	0.99
Lactoferrin	0.72 b	0.76 b	0.86 a	0.012	4.40	0.84	0.72	0.001	11.00	0.71	0.85	<0.001	0.71	0.238	1.44	0.564	0.57
Resveratrol	0.5	0.53	0.56	0.319	1.14	0.51	0.55	0.229	1.45	0.42	0.64	<0.001	0.42	0.038	3.28	0.119	2.13
Quercetin	0.49	0.53	0.57	0.182	1.70	0.53	0.53	0.853	0.03	0.41	0.65	<0.001	0.41	0.283	1.26	0.405	0.90

a, b, c: Means with different letters correspond to statistical differences (*p* < 0.05).

**Table 8:** Contingency tables used to summarize the relationship between bioactive compounds’ efficacy against COVID-19, according to FIS group.

Substance	Efficacy	FIS Score							
		Low		Medium		High		All	
		*n*	%	*n*	%	*n*	%	*n*	%
Fibers	Effective	67	7.10	72	8.50	97	9.90	236	8.50
	Not effective	373	39.60	314	37.10	375	38.10	1062	38.30
	Do not know	503	53.30	461	54.40	511	52.00	1475	53.20
Vitamins *	Effective	253	26.80	272	32.10	336	34.20	861	31
	Not effective	248	26.30	194	22.90	211	21.50	653	23.50
	Do not know	442	46.90	381	45.00	436	44.40	1254	45.90
Mineral salts	Effective	109	11.60	100	11.80	133	13.50	342	12.30
	Not effective	325	34.50	285	33.60	308	31.30	918	33.10
	Do not know	509	54.00	462	54.50	542	55.10	1513	54.60
Supplements	Effective	154	16.30	125	14.80	191	19.40	470	16.90
	Not effective	309	32.80	264	31.20	301	30.60	874	31.50
	Do not know	480	50.90	458	54.10	491	49.90	1429	51.50
Probiotics *	Effective	90	9.50	111	13.10	165	16.80	366	13.20
	Not effective	266	28.20	223	26.30	261	26.60	750	27
	Do not know	587	62.20	513	60.60	557	56.70	1657	59.80
Fermented cereals	Effective	47	5.00	37	4.40	47	4.80	131	4.70
	Not effective	333	35.30	272	32.10	333	33.90	938	33.80
	Do not know	563	59.70	538	63.50	603	61.30	1704	61.40
Prebiotics *	Effective	74	7.80	83	9.80	128	13.00	285	10.30
	Not effective	259	27.50	197	23.30	254	25.80	710	25.60
	Do not know	610	64.70	567	66.90	601	61.10	1778	64.10
Polyphenols	Effective	66	7.00	62	7.30	90	9.20	218	7.90
	Not effective	223	23.60	188	22.20	223	22.70	634	22.30
	Do not know	654	69.40	597	70.50	670	68.20	1921	69.30
Vitamin C	Effective	284	30.10	295	34.80	379	38.60	958	34.50
	Not effective	243	25.80	189	22.30	213	21.70	645	23.30
	Do not know	416	44.10	363	42.90	391	39.80	1170	42.20
Vitamin D	Effective	272	28.80	285	33.60	346	35.20	903	32.60
	Not effective	235	24.90	188	22.20	223	22.70	646	23.30
	Do not know	436	46.20	374	44.20	414	42.10	1224	44.10
Vitamin A	Effective	130	13.80	111	13.10	153	15.60	394	14.20
	Not effective	274	29.10	222	26.20	242	24.60	738	26.60
	Do not know	539	57.20	514	60.70	588	59.80	1641	59.20
Green Tea	Effective	55	5.80	50	5.90	78	7.90	183	6.60
	Not effective	390	41.40	335	39.60	385	39.20	1110	40.00
	Do not know	498	52.80	462	54.50	520	52.90	1480	53.40
Propolis	Effective	93	9.90	91	10.70	142	14.40	326	11.80
	Not effective	320	33.90	276	32.60	317	32.20	913	32.90
	Do not know	530	56.20	480	56.70	524	53.30	1534	55.30
Melatonin	Effective	53	5.60	41	4.80	51	5.20	145	5.20
	Not effective	390	41.40	337	39.80	400	40.70	1127	40.60
	Do not know	500	53.00	469	55.40	532	54.10	1501	54.10
Curcumin	Effective	70	7.40	55	6.50	86	8.70	211	7.60
	Not effective	288	30.50	224	26.40	282	28.70	794	28.60
	Do not know	585	62.00	568	67.10	615	62.60	1768	63.80
Lactoferrin *	Effective	86	9.10	79	9.30	109	11.10	274	9.90
	Not effective	241	25.60	167	19.70	205	20.90	613	22.10
	Do not know	616	65.30	601	71.00	669	68.10	1886	68
Resveratrol	Effective	32	3.40	34	4.00	49	5.00	115	4.1
	Not effective	201	21.30	147	17.40	180	18.30	528	19
	Do not know	710	75.30	666	78.60	754	76.70	2130	76.80
Quercetin	Effective	46	4.90	47	5.50	61	6.20	154	5.60
	Not effective	259	27.50	197	23.30	254	25.80	527	19
	Do not know	700	74.20	652	77.00	740	75.30	2092	75.40

Association between efficacy and FIS group for each bioactive compounds was analyzed by chi-square test (* *p* < 0.05).

**Table 9:** Bioactive compound efficacy against COVID-19, according to consumers’ consensus, versus the number of publications related to efficacy against COVID-19 in the Web of Science Platform (WOS) using the search “name of each compound” and “COVID”.

Substance	Efficacy (%)	Number of Publications
Vitamin A	14.2	1691
Vitamin D	32.6	1419
Vitamin C	34.5	839
Probiotics	13.2	241
Supplements	16.9	216
Polyphenols	7.9	193
Curcumin	7.6	184
Melatonin	5.2	178
Micronutrients	12.3	94
Green tea	6.6	84
Fibers	8.5	56
Quercetin	5.6	46
Propolis	11.8	42
Vitamins	31	38
Fermented food	4.7	27
Resveratrol	4.1	19
Lactoferrin	9.9	15
Prebiotics	10.3	11

## Data Availability

The data presented in this study are available on request from the corresponding author.
